# Evaluating the effects of the Lunchtime Enjoyment Activity and Play (LEAP) school playground intervention on children’s quality of life, enjoyment and participation in physical activity

**DOI:** 10.1186/1471-2458-14-164

**Published:** 2014-02-14

**Authors:** Brendon P Hyndman, Amanda C Benson, Shahid Ullah, Amanda Telford

**Affiliations:** 1 Discipline of Exercise Sciences, School of Medical Sciences, RMIT University, Melbourne, Australia; 2 Flinders Centre for Epidemiology and Biostatistics, School of Medicine, Flinders University, Adelaide, Australia

**Keywords:** Physical activity, Primary school, Intervention, Lunchtime, Children, Enjoyment, Quality of life, Recess, School playgrounds

## Abstract

**Background:**

An emerging public health strategy is to enhance children’s opportunities to be physically active during school break periods. The aim of this study was to evaluate the effects of the Lunchtime Enjoyment Activity and Play (LEAP) school playground intervention on primary school children’s quality of life (QOL), enjoyment and participation in physical activity (PA).

**Methods:**

This study consisted of a movable/recycled materials intervention that included baseline, a 7-week post-test and an 8-month follow-up data collection phase. Children within an intervention school (n = 123) and a matched control school (n = 152) aged 5-to-12-years-old were recruited for the study. Children’s PA was measured using a combination of pedometers and direct observation (SOPLAY). Quality of life, enjoyment of PA and enjoyment of lunchtime activities were assessed in the 8-12 year children. A multi-level mixed effect linear regression model was applied in STATA (version 12.0) using the *xtmixed* command to fit linear mixed models to each of the variables to examine whether there was a significant difference (p < 0.05) between the intervention and control school at the three time points (pre, post and follow-up).

**Results:**

Significant overall interaction effects (group × time) were identified for children’s mean steps and distance (pedometers) in the intervention school compared to the control school. Intervention school children also spent significantly higher proportions within specified target areas engaged in higher PA intensities in comparison to the control school at both the 7-week post-test and 8-month follow-up. A short-term treatment effect was revealed after 7-weeks for children’s physical health scale QOL, enjoyment of PA and enjoyment of intra-personal play activities.

**Conclusions:**

Examining the effects of this school playground intervention over a school year suggested that the introduction of movable/recycled materials can have a significant, positive long-term intervention effect on children’s PA. The implications from this simple, low-cost intervention provide impetus for schools to consider introducing the concept of a movable/recycled materials intervention on a wider scale within primary school settings.

**Trial registration:**

Australian and New Zealand Clinical Trials Registration Number: ACTRN12613001155785.

## Background

The promotion of physical activity (PA) in society has become a significant public health priority to enhance health worldwide and prevent chronic diseases such as type two diabetes, obesity and cardiovascular disease [1]. In Australia, similar to other countries [2], 31% of Australian children are not meeting national guidelines for PA [3]. Despite childhood being an important period to establish regular PA patterns that can track across the lifespan [4], our understanding of strategies to develop and sustain health enhancing PA behaviours among school children is limited [1,5].

The school environment has been established as one of the most important settings to facilitate children’s PA [6,7], particularly as children spend significant portions of their day at or in transit to and from school [8]. A reduction in children’s PA opportunities [9] and the growth of overweight and obese youth worldwide [10] has placed schools at the forefront of preventative public health as a key setting to develop children’s PA. With growing attention on schools to offer PA opportunities, there is a need to provide children with the essential skills to be physically active [11]. Despite this attention, research has identified a number of barriers to the delivery of effective Physical Education (PE) in schools [12]. With the many demands and responsibilities placed upon PE teachers [12], it is important to explore other avenues within the school setting to facilitate PA [13]. Children’s diverse learning needs and personalities may also respond to a range of non-curricular opportunities that facilitate PA [14].

### Moving school physical activity beyond structured physical education

A key area of school-based PA research that has gained momentum is the implementation of strategies during school breaks [13,15]. Beyond school breaks, children may have limited access to PA opportunities [16], therefore providing active play opportunities that can be replicated within the home and community settings could produce many health benefits [17,18]. Active play is regarded as the diverse range of unstructured activities and behaviour that children engage in [19]. Active play has been acknowledged as the ‘informal curriculum’ [19] to facilitate children’s learning and development, generating a widespread international pursuit to improve school playgrounds to optimise children’s play [20]. Active play has also been acknowledged by the United Nations High Commission for Human Rights as a basic entitlement for every child [21]. Children’s active play opportunities during school breaks require little organisational input and instruction from teachers and parents. Children in many schools are engaging in up to 600 school break periods per year (3 times per day, 5 days per week, 39 weeks per year) [22]. School breaks offer substantial time and opportunity for children to be physically active. Primary school children aged 5-12 years are estimated to spend at least 30 hours per week attending school and can accumulate up to 35% of their active play during school breaks engaged in moderate to vigorous physical activity (MVPA) [23]. Additionally, active play during break periods has been recognised as the primary source of children’s PA [24], contributing up to 50% of children’s recommended daily PA [24-27], improvements in classroom behaviour [27] and development of social and physical skills [28]. Active play has also been reported to enhance children’s coping skills and has been suggested to promote psychological wellbeing by fostering intrinsic motivation, competence and a sense of belonging [28]. With approximately 14% of Australian children experiencing mental health problems [28], maximising quality play opportunities during school breaks has the potential to enhance children’s physical and mental health.

### Targeting school break periods to encourage physical activity and active play

Whilst a well-designed school environment can enhance children’s physical and mental health, Australian data reveals many schools have eliminated play spaces and equipment, have crowded play spaces and implement restrictive play policies (e.g. reduced playground access, over-policing of safety rules), resulting in fewer opportunities for children to experience active play [29,30]. A number of interventions targeting school breaks have successfully attempted to counteract this decline in children’s PA by implementing active supervision [31], school break periods with a weekly activity theme [32], the provision of sports or games equipment and activity cards [25,33], fitness breaks [34-36], school playground markings [22,37] and physical playground structures [38] to facilitate children’s PA. These interventions generally foster structured PA with specified locations, time schedules, adult supervision [39] and the facilitation of sport and fitness [39]; there is a need to examine school break interventions that encourage unstructured play [40-42].

Unstructured PA is defined as the PA children participate in that is spontaneous and without a set regime or purpose [43] that can include digging, raking [44], lifting/carrying, exploring, planting, chasing [41], pushing objects into positions, construction, imaginative and creative play [45]. The importance of children’s unstructured PA is reflected in the definition of school breaks, “as a regularly scheduled time for children to engage in ‘unstructured’ PA and play” (p123) [46]. Introducing natural environmental features [42,47], play pods [48] and movable/recycled materials [45,49,50] are examples of unstructured interventions that can be used during school breaks that have provided diversity to children’s play, developing playfulness (e.g. intrinsic activity without a set regime or purpose) physical, cognitive and social outcomes and appeal to a broad range of children.

The effects of introducing movable/recycled materials have been reported after a small pilot (n = 12) [45] and larger trial (n = 226; 12 schools) [50] in children aged 5-7-years-old via the use of a playfulness measure [49], a single PA measure (accelerometers) and teacher interviews [45]. The positive increases in PA and playfulness reported in the pilot and larger trial studies demonstrate the potential to examine a movable/recycled materials intervention targeting a whole school (5-12-years-old) with the measurement of additional health and PA outcomes. There is also a need to increase our understanding of the mediators on children’s PA within school settings [51] (e.g. enjoyment), as well as long-term intervention effects [51]. No study we are aware of has examined the influences of a school playground intervention on children’s quality of life (QOL) and there is a need to examine the effectiveness of interventions targeting school breaks underpinned by the social-ecological model [52].

### Aims

The aim of this study was to examine the effects of the Lunchtime Enjoyment Activity and Play (LEAP) movable/recycled materials school playground intervention on primary school children’s QOL, enjoyment and participation in PA.

## Methods

### Study design

**Figure 1:**
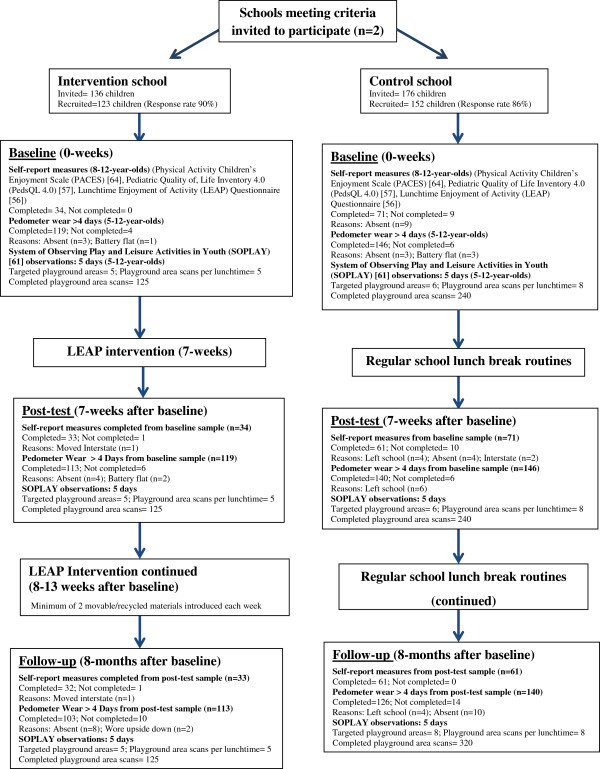
Flow of LEAP intervention recruitment, measures and responses.

This matched controlled trial, the LEAP intervention study, was uniquely tailored to compare the intervention and control schools at baseline (March/April, 2010), post-testing (after 7-weeks; April-June, 2010) and at a follow-up (after 8-months; November, 2010) (Figure 1). The intervention provided movable/recycled materials for children to use in the school playground with usual playground supervision by teachers (yard duty). Children in the control school continued their PA with their usual sports equipment, fixed playground equipment and teacher supervision.

### Participants and recruitment

All children within each primary school (aged 5-12-years-old) received a plain language statement outlining the research, along with a participant and parental consent form. A total of 123 children from the intervention school (mean 7.0 years ±1.9; 90% response rate) and 152 children from the control school (mean 8.2 years ±2.1; 86% response rate) returned signed informed parental consent forms to participate in the study (Figure 1). Ethical approval for the study was obtained from the University of Ballarat Human Research Ethics Committee, the Catholic Education Office of the Archdiocese of Ballarat and permission was gained from the school principals.

### School selection

A newly developed catholic co-educational primary school with no fixed playground equipment was approached to participate as the intervention school. A control school matched by sector, school type (co-education, prep to year 6; 5-12-year-olds, socio-economic status, size of school grounds and enrolment) was selected and recruited to participate in the study via emails, phone calls and on-site visits to the Principal. Both schools were located in the same geographical area in Regional Western Victoria, Australia. All children in the study participated in their regular daily school routines.

### Intervention school playground environment

During the LEAP intervention, mean (SD) maximum temperatures during baseline, post-test and follow-up at the intervention school were 23.25 (±4.68°C), 14.88 (±2.06°C) and 21.36 (±4.83°C) respectively. The LEAP intervention, which included movable/recycled materials, was designed based upon the social-ecological model; which emphasises that intra-personal, inter-personal, physical environment/policy levels may all influence behaviour [52]. Table 1 reports how the social-ecological model underpinned the design of the present study, depicting how the levels of influence on children’s health behaviour were measured.

**Table 1:** Assessment of social-ecological model levels of influence during the LEAP intervention

				**Data collection timeframe**		
**Social-ecological model levels**[52]	**Outcome variable**	**Measurement tool**	**Data collection method**	**Baseline**	**Post-test (7-weeks)**	**Follow-up (8-months)**
**Intra-personal (Individual) level factors**	• Individual level physical activity duration, frequency, steps and distances during school lunch breaks.	• Pedometers	• Worn by children on the right hip	• 5 days	• 5 days	• 5 days
	• Enjoyment of general physical activity.	• PACES	• Child self-report	• 1 occasion	• 1 occasion	• 1 occasion
	• Enjoyment of intra-personal related play activities.	• LEAP Questionnaire	• Child self-report	• 1 occasion	• 1 occasion	• 1 occasion
	• Physical health domain score of quality of life.	• Peds QL 4.0	• Child self-report	• 1 occasion	• 1 occasion	• 1 occasion
**Inter-personal (Social) level factors**	• Area-level physical activity intensities over the school year.	• SOPLAY	• Video	• 5 days	• 5 days	• 5 days
	• Enjoyment of inter-personal-related play activities.	• LEAP Questionnaire	• Child self-report	• 1 occasion	• 1 occasion	• 1 occasion
	• School, social and home-related quality of life as a result of the intervention (within psychosocial and overall quality of life domains).	• Peds QL 4.0	• Child self-report	• 1 occasion	• 1 occasion	• 1 occasion
**Physical environment/policy level factors**	• The physical activity types children participated in within the school playground during school lunch breaks.	• SOPLAY	• Video	• 5 days	• 5 days	• 5 days
	• Enjoyment of physical environment/policy-related play activities.	• LEAP Questionnaire	• Child self-report	• 1 occasion	• 1 occasion	• 1 occasion

SOPLAY [61] = System of Observing Play and Leisure Activities in Youth; LEAP [56] = Lunchtime Enjoyment of Activity and Play; PedsQL 4.0 [57] = Pediatric Quality of Life Inventory; PACES [64] = Physical Activity Children’s Enjoyment Scale; LEAP questionnaire consisted of 39 items (Intra-personal level component: 20 items; Inter-personal level component: 2 items; Physical Environment/Policy level component: 17 items); PACES consisted of 16 scale items; Peds QL consisted of 23 items (Psychosocial health scale quality of life: 15 items; Physical health scale quality of life: 8 items).

Movable/recycled materials with no fixed purpose were introduced to a grass field in a brand new Catholic primary school from the end of term 1 to the middle of term 2 (after 7-weeks: post-test) and continued to be introduced until the end of term 2 during Autumn and Winter in 2010 (Figure 1). As the school grounds were brand new, there was only one other play area, a car-park area which was commonly used during wet conditions or for those children not interested in playing on the field. There was no fixed play equipment in the school grounds during the intervention (e.g. climbing frames, monkey bars, slides). The movable/recycled materials introduced to the playground by the researchers were items generally not considered to be typical play materials for children within schools, with the exception of play balls, hoops and skipping ropes. The materials included milk crates, swimming noodles, buckets, cardboard boxes, tyre tubes, pipes, vacuum/pool hoses, plastic walls and sheets, hessian bags, buckets, water/sand shells, tractor/motorbike and bicycle tyres, swimming boards, exercise mats, buckets and hay bales. Five materials were introduced during the first week of the program, and each week thereafter a maximum of two additional types of material were introduced during the intervention period to avoid over-stimulation. All items remained on the field after being added, except for the removal or replacement of items that were broken or if teachers perceived an item presented a safety issue.

The grass field at the intervention school was of triangular shape and a steep incline, with each boundary 95 m (bottom) × 105 m (top) × 90 m (left side) bordered by trees and bushes on the bottom and left boundaries. The top boundary was bordered by a main road. Near the entrance beyond the left side border was a rectangular stretch of grass 50 m × 20 m on a downward incline which was considered out of bounds. Conforming to Australian/New Zealand Safety Standards [53], children were instructed to not stack more than two hay bales on top of each other, which was considered notionally the same as waist height. In addition, teachers instructed the students that only the research team and teaching staff could move the tractor tyres to other parts of the grass field, children were not permitted to strike each other with the swimming noodles and children had to return all equipment at the end of the week to the entrance of the grass field.

Children were on the playground for 30 minutes at morning break and 30 minutes during the lunchtime period. All students (5-12-year-olds) had access to the playground simultaneously. The provision of small pieces of portable sports equipment was made available by the school such as footballs, bats and balls as per usual practice in primary schools. Two teachers were rostered on school playground supervision (yard duty) during school breaks as was usual practice, one teacher was allocated to supervise the grass field and the other to supervise the bitumen car park area. The principal briefed the teachers prior to students commencing the intervention, explaining that the items were to encourage children to create their own play and not to intervene unless children’s safety was at risk.

### Control school playground environment

The Control School did not have access to the movable/recycled materials used with the intervention school and consisted of a morning break of 15 minutes and a lunchtime break of 45 minutes. Children had access to sports equipment as is usual practice in many primary schools to use on the hard-surfaced area at the front of the school and on the grass field during break periods. Mean (SD) maximum temperatures during baseline, post-test and follow-up at the control school were 22.86 (±5.96°C), 12.76 (±2.37°C) and 16.54 (±3.92°C) respectively (Table 2). The control school’s playground area consisted of a 10 m × 70 m bitumen area stretch alongside the school buildings at the front of the school with playground markings (for hopscotch and down-ball type activities). Also at the front of the school alongside the hard-surfaced area was a 10 m × 10 m area of rocks and a 37 m × 17 m area that included three built playgrounds with wooden bridges, climbing frames, monkey bars, ladders and slides. Connecting the front of the school to the school’s 75 m × 70 m grassed oval was a 23 m walkway. The control school’s grass field consisted of a set of Australian Rules Football and soccer goal posts and was surrounded by a line of tall trees, a spider web playground structure and a large sandpit. Beyond the control school’s grass field was a 34 m × 36 m basketball court area. Two teachers supervised the playground during lunchtime at all times (one supervisor was allocated to the fixed playground area at the front of the school, the other teacher supervised the grass field and basketball court areas at the bottom of the school).

**Table 2:** Baseline demographic variables, objective and self-report measures of quality of life, enjoyment and physical activity

**Baseline characteristics**		**Intervention school**	**Control school**	***p***^**1**^
		**(n = 123)**	**(n = 152)**	
**Demographics**				
Boy (%)		53.7	46.7	0.05
Age (Years) (Mean (SD))		7.0 (1.9)	8.2 (2.1)	<0.001
Age (Years) (%)				<0.001
5-7		65.0	40.6	
8-9		20.3	31.4	
10-12		14.6	28.1	
**Objective measures of physical activity**				
Pedometer Mean (SD)	Steps per minute	62.2 (20.2)	53.0 (17.2)	<0.001
	Distance per minute (metres)	41.9 (17.1)	38.8 (15.3)	0.14
**Self-reported measures**				*p*^2^
PEDS QL 4.0 [57] Median (IQR)	Physical health scale quality of life	78.1 (62.5-90.6)	87.5 (75.0-93.8)	<0.001
	Psychosocial scale quality of life	73.3 (61.7-85.0)	78.3 (68.3-88.3)	0.20
	Overall quality of life	76.9 (62.1-85.8)	83.4 (70.8-90.8)	0.04
PACES Survey [64] Median (IQR)	Enjoyment of physical activity	4.5 (4.2-4.9)	4.5 (4.1-4.8)	0.38
LEAP Questionnaire [56] Median (IQR)	Intra-personal level enjoyment	4.3 (3.8-4.6)	4.1 (3.7-4.5)	0.31
Inter-personal level enjoyment	5.0 (4.5-5.0)	5.0 (4.5-5.0)	0.59
	Physical environment/policy level enjoyment	4.1 (3.8-4.4)	4.1 (3.7-4.5)	0.95

^1^*p* values are based on independent sample *t*-test for continuous normal data and Chi-square test for categorical data; ^2^*p* values are based on Mann Whitney U test for non-normal data; IQR = Interquartile Range; SD = Standard Deviation; % = Percentage.

### Intervention outcome measures

The primary outcome variable of the LEAP intervention was PA, individually and objectively measured by pedometers in children aged 5-12-years-old. In addition, the System of Observing Play and Leisure Activities in Youth (SOPLAY), an area-level direct observation instrument was used to provide contextual information on the children’s PA within the school playground [54]. The secondary outcome variables included enjoyment of PA [55], enjoyment of lunchtime play activities [56] and QOL [57] in those children aged 8-12-years-old.

### Pedometers

Children’s steps and distance were assessed using a Yamax Digiwalker SW200 pedometer (the monitor was taped closed to prevent tampering during the lunchtime breaks). The Yamax Digiwalker pedometer has been validated for measurement in children within laboratory and field settings [54,58]. On the initial day of monitoring, children were instructed on how to wear the pedometer (attachment on the right hip) and the pedometer’s removal (immediately after school lunchtime breaks). Children were asked to wear the pedometer during the whole of school lunchtime breaks and instructed to place the monitor into a storage box at the conclusion of lunchtime breaks as they were lining up to enter their classroom. The investigators and class teacher ensured that no child was still wearing a pedometer. The total step counts for each individual child were recorded immediately after school lunchtime breaks into a Microsoft Excel version 14.0 (Windows Corporation, 2010) spread-sheet. Researchers recorded if the child’s pedometer battery went flat, the child was absent or if the pedometer was faulty. Pedometer counts were converted to steps per minute by dividing total steps by the number of lunchtime minutes to ensure school lunchtime break length differences were accounted for between the two schools. For a number of reasons (e.g. child forgetting to wear the pedometer, student was absent, battery was flat) full pedometer data were not available for all children for all lunchtime break periods (Figure 1).

To calculate children’s stride length, children were instructed to walk one at a time across a flat surfaced area of the school playground twice over a 20 metre distance. Investigators counted and recorded the steps it took the child to walk the 20 metres and the mean steps from the two trials were calculated. The stride length was calculated by dividing the total distance walked (20 metres) by the mean step count [59]. Measuring stride length allowed for the calculation of total distance (metres) of PA during the data collection phases to be calculated by using the following formula; stride length × steps = distance (metres) [60].

### Direct observation: SOPLAY

Area-level PA intensities, PA types and the context for play were measured using the System of Observing Play and Leisure Activities in Youth (SOPLAY) [61]. Training of assessors included familiarisation with the protocol and codes (activity codes were modified to include imaginative play with and without movable/recycled materials) and practicing observations using video examples of school breaks. Lunchtime video recordings were conducted for five days during each data collection phase. Video cameras captured each defined target area within the playgrounds of the intervention and control schools. Video facilitated direct observation is suggested to increase reliability of direct observation measurement [62]. All school playground target areas were identified prior to PA measurement by determining key areas in which play generally took place. No indoor observations were conducted during the study. Investigators and research assistants provided commentary to assist in activity coding and ensured each video camera was unimpeded from capturing school playground footage. After consultation with the SOPLAY designer Thom Mckenzie, it was decided that capturing video would allow the original lunchtime scanning protocol of two scans (scan one: 15 minutes after the commencement of lunchtime; scan two: 10 minutes after scan one) to be increased to scanning at five minute intervals (5 × scans over 30 minutes: intervention school; 8 × scans over 45 minutes: control school) during school lunchtime breaks to increase the sensitivity of the instrument over a lunchtime-specific data collection period. The videos were transferred to computers using the iMovie 2011™ (Apple Inc., 2011) software and stored. After the transfer, the captured data were coded using the SOPLAY instrument. Due to Australia having high levels of skin cancer [63] both schools had a policy for skin protection of ‘No Hat, No Play’. This meant it was not possible to determine the sex-specific identification of children during the PA scans from all video recordings and therefore this sub-categorisation was not captured.

### Enjoyment of physical activity (8-12-year-olds)

The Physical Activity Children’s Enjoyment Scale (PACES) was used to determine children’s general enjoyment of PA. The revised PACES is reliable [64] and comprehensive [65] for school-aged children aged 8-years-old and over, consisting of a 16 statement scale starting with the question stem ‘When I am physically active…” with a 5-point likert scale (1 = disagree a lot; 2 = disagree; 3 = no opinion; 4 = agree; 5 = agree a lot). A score is computed by calculating the mean of the 16 items [64].

### Enjoyment of school play activities (8-12-year-olds)

The Lunchtime Enjoyment of Activity and Play (LEAP) Questionnaire was used to measure children’s enjoyment of school play activities [56]. The LEAP questionnaire is a reliable, context-specific questionnaire consisting of 39 items, categorised by social-ecological model levels (intra-personal, inter-personal, physical environment/policy) to identify the broader influences on children’s enjoyment of school play and lunchtime activities [56]. All enjoyment items were rated on a five-point likert scale (1 = very unhappy; 2 = unhappy; 3 = not sure; 4 = happy; 5 = very happy) [56]. A score is computed by calculating the average of each social-ecological model component.

### Quality of Life (8-12-year-olds)

The Pediatric Quality of Life Inventory 4.0 (PedsQL), a 23-item validated questionnaire was used to measure the QOL in children aged 8-12-years-old [57]. The PedsQL instrument measures QOL in three scales; psychosocial, physical and total QOL. The PedsQL has been established as reliable for use with school children as young as 8-years-old [57]. The questionnaire is scored using a five-point likert scale (0 = never; 1 = almost never; 2 = sometimes; 3 = almost always; 4 = always), with items then converted to a score out of 100 (0 = 100; 1 = 75; 2 = 50; 3 = 25; 4 = 0). A mean score is calculated for the psychosocial and physical QOL scales. The scales are averaged to obtain a total QOL score [57].

### Field notes

Descriptive accounts of the LEAP intervention during the course of collecting or reflecting on the data were recorded via field notes [66,67]. The field notes were used to complement the objective and self-report instruments by recording what could be seen, heard, experienced and thought of [66,67] during children’s engagement with the movable/recycled materials. The investigators minimised any influence on the setting by positioning in unobtrusive positions along the boundary of the school playgrounds and randomly recording children’s PA behaviour [67].

### Data analysis

A Chi-square test was used to determine significant differences between the intervention and control schools for demographic characteristics. An independent sample *t*-test was used to determine significant differences between the intervention and control schools for baseline objective measurements (p < 0.05 was significant). A non-parametric Mann-Whitney *U* test was used to determine the significant difference between ranks for each self-reported measure between the intervention and control schools (p < 0.05 was significant). A multi-level mixed effect linear regression model was applied using STATA (version 12.0) using the *xtmixed* command to fit linear mixed models for pedometer steps and distance per minute, enjoyment of PA, enjoyment of lunchtime play activities and QOL. The model was used to determine the treatment effects (adjusted mean change in the intervention school compared to the control school at each time point) and interaction effects (overall effects on the intervention school compared to the control school for all three time points: pre, post and follow-up). As the scores changed with age and sex, the model was adjusted by age and sex. The models were also adjusted by baseline measurements (as a function of the linear regression model) as significant differences were found at baseline between schools. A Chi-square test was used to compare the direct observation proportions in each PA intensity and activity type between intervention and control schools. Content analysis of field notes was based upon identifying emerging themes relating to children’s uptake and use of the movable/recycled materials for PA. Missing data for a time point (e.g. missing questionnaire responses) were excluded pairwise from the analyses.

## Results

### Physical activity

**Figure 2:**
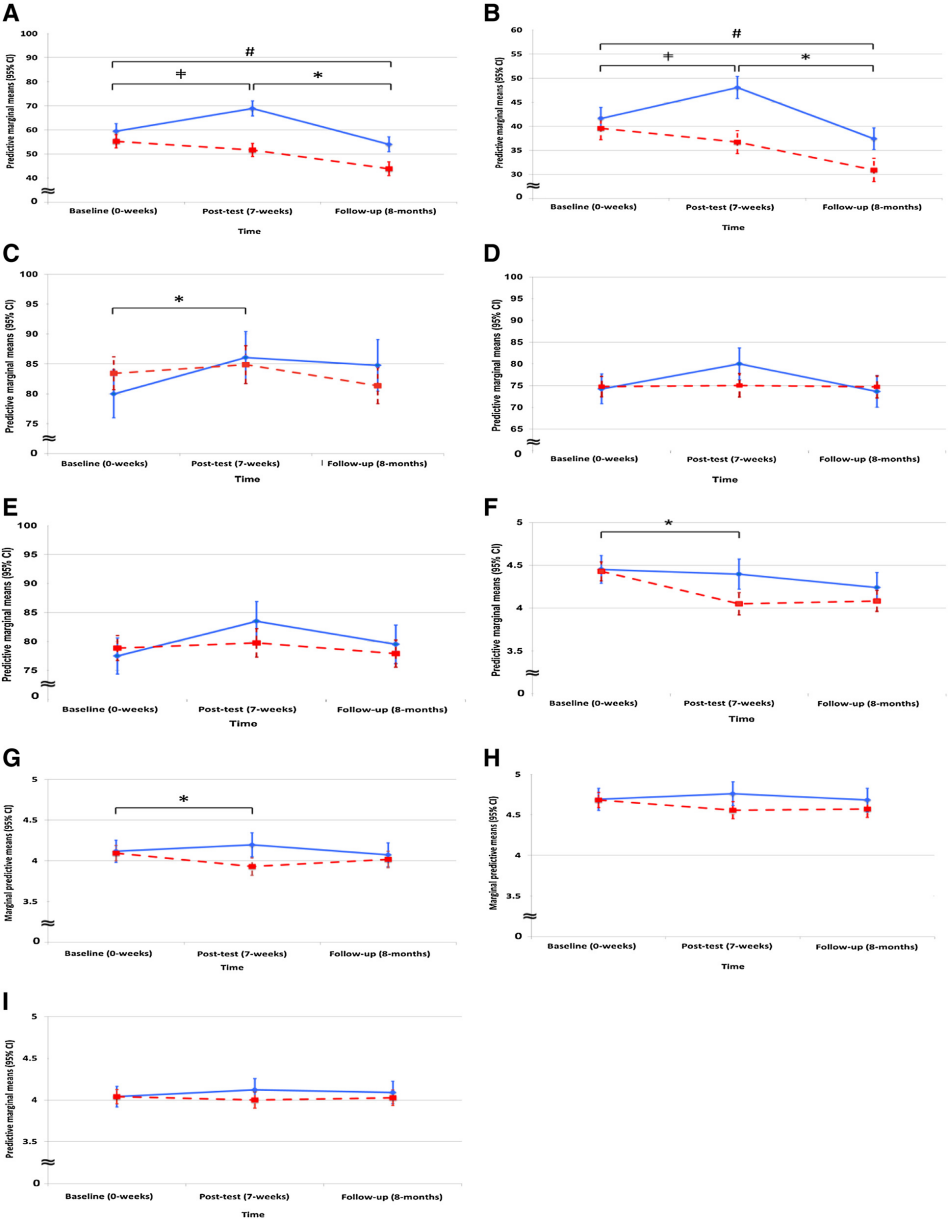
**Adjusted means of outcome measures for intervention and control schools at baseline, post-test and follow-up. (A)** Pedometer steps per minute; **(B)** Pedometer distance per minute (metres); **(C)** Physical health scale quality of life; **(D)** Psychosocial health scale quality of life; **(E)** Overall quality of life; **(F)** Enjoyment of physical activity; **(G)** Enjoyment of intra-personal level play activities; **(H)** Enjoyment of inter-personal level play activities; **(I)** Enjoyment of physical environment/policy level play activities; Model adjusted by age, sex and baseline measurements; ╪= Significant treatment effect, *p* < 0.001; * = Significant treatment effect, *p* < 0.05; # = Significant overall interaction effect, *p* < 0.001; 95% CI = Confidence interval; “Blue line”= Intervention school, “Red broken line”= Control school.

A significant treatment effect was identified from the multi-level linear regression model for the intervention school children’s pedometer-determined mean steps per minute in comparison to the control school from baseline to the 7-week post-test (+13.08 adjusted mean steps per minute, 95% CI 7.31-18.84, p < 0.001) and from baseline to the 8-month follow-up (+5.93 adjusted mean steps per minute, 95% CI 0.14-11.72, p = 0.045). Similarly, a significant treatment effect was also identified for the intervention school children’s distance per minute in comparison to the control school from baseline to the 7-week post-test (+9.32 adjusted mean metres per minute, 95% CI 4.82-13.82, p < 0.001) and from baseline to the 8-month follow-up (+4.47 adjusted mean metres per minute, 95% CI -0.02-9.96, p = 0.051) (Table 3 and Figure 2). However, the increments were lower during follow-up than post-test for both steps and distance. A significant overall interaction effect was identified for both steps and distance per minute in the intervention school compared to the control school for the three time points (Table 3 and Figure 2).

**Table 3:** Multi-level linear regression model of measures between intervention and control schools at baseline, post-test and follow-up from the LEAP intervention

**Measurement tool**	**Category**	**Time**	**Intervention (n = 123)**	**Control (n = 152)**	**Treatment effect**^#^	***p *** **value**	***p *** **value**
			**Δ (95% CI)**	**Δ (95% CI)**		**(treatment effect)**	**(overall interaction effect)**
**Objective measures**							
**Pedometer**	Steps per minute	Baseline					<0.001
		Post-test	9.48 (5.17-13.78)	-3.60 (-7.43-0.24)	13.08 (7.31-18.84)	<0.001	
		Follow-up	-5.44 (-9.76--1.12)	-11.37 (-15.26--7.48)	5.93 (0.14-11.72)	0.05	
	Distance per minute (metres)	Baseline					<0.001
		Post-test	6.44 (3.34-9.55)	-2.88 (-6.13-0.38)	9.32 (4.82-13.82)	<0.001	
		Follow-up	-4.22 (-7.34--1.09)	-8.69 (-11.95--5.42)	4.47 (-0.02-9.96)	0.05	
**Self-report measures**							
**PEDS QL 4.0 **[57]	Physical health scale of quality of life	Baseline					0.14
		Post-test	6.07 (0.36-11.77)	1.46 (-2.66-5.57)	4.61 (-2.42-11.64)	0.20	
		Follow-up	4.76 (-1.02-10.54)	-2.08 (-6.10-1.94)	6.84 (-0.10-13.78)	0.05	
	Psychosocial scale of quality of life	Baseline					0.09
		Post-test	5.74 (1.13-10.35)	0.28 (-3.05-3.61)	5.46 (-0.22-11.14)	0.06	
		Follow-up	-0.59 (-5.27-4.09)	-0.08 (-3.34-3.18)	-0.51 (-6.11-5.09)	0.86	
	Overall quality of life	Baseline					0.17
		Post-test	6.00 (1.67-10.34)	0.90 (-2.23-4.03)	5.10 (-0.24-10.45)	0.06	
		Follow-up	2.01 (-2.38-6.41)	-0.95 (-4.01-2.11)	2.96 (-2.31-8.23)	0.27	
**PACES Survey **[64]	Enjoyment of physical activity	Baseline					0.09
		Post-test	-0.06 (-0.29-0.18)	-0.38 (-0.54--0.21)	0.32 (0.04-0.61)	0.03	
		Follow-up	-0.21 (-0.45-0.02)	-0.34 (-0.51--0.18)	0.13 (-0.15-0.41)	0.36	
**LEAP Questionnaire **[56]	Intra-personal level enjoyment	Baseline					0.11
		Post-test	0.08 (-0.11-0.28)	-0.16 (-0.30--0.02)	0.24 (0.004-0.48)	0.05	
		Follow-up	-0.04 (-0.24-0.16)	-0.08 (-0.21-0.06)	0.03 (-0.20-0.27)	0.78	
	Inter-personal level enjoyment	Baseline					0.23
		Post-test	0.07 (-0.11 (0.25)	-0.13 (-0.26-0.01)	0.20 (-0.03-0.42)	0.09	
		Follow-up	-0.01 (-0.20-0.18)	-0.11 (-0.24-0.02)	0.10 (-0.12-0.33)	0.37	
	Physical environment/policy level enjoyment	Baseline					0.52
		Post-test	0.08 (-0.09-0.26)	-0.04 (-0.17-0.08)	0.12 (-0.09-0.34)	0.26	
		Follow-up	0.05 (-0.13-0.23)	-0.01 (-0.14-0.11)	0.06 (-0.14-0.27)	0.57	

PEDS QL 4.0 [57] = Paediatric Quality of Life Inventory; PACES [64] = Physical Activity Children’s Enjoyment Scale; LEAP [56] = Lunchtime Enjoyment of Activity and Play; Δ = Adjusted mean change between baseline and post intervention from the multi-level linear regression model; Model adjusted by age, sex and baseline measurements; ^#^Effects in intervention school compared with the control school after adjustment for age, sex and baseline measures from the multi-level linear regression model; 95% CI = Confidence interval.

Direct observation comparisons from the Chi-square statistical test identified no significant differences in the area-level PA between schools during lunchtime breaks at baseline for the proportion of children in sedentary behaviour, moderate intensity PA (MPA) and vigorous intensity PA (VPA). After the LEAP intervention was introduced, the mean proportion of children observed at the intervention school participating in VPA was significantly higher than the control school (7-week post-test: +6.2% mean proportion of observed children, p = <0.01; 8-month follow-up: +6.2% mean proportion of observed children, p = 0.01) and the mean proportion of children observed participating in sedentary behaviour was significantly less than the control school (7-week post-test: -5.6% mean proportion of observed children, p = <0.01; 8-month follow-up: -15.2% mean proportion of observed children, p = <0.001).

There was no significant difference in the mean proportion of children observed participating in moderate physical activity (MPA) between schools at the 7-week post-test, however the mean proportion of children participating in MPA was significantly higher in the intervention school at the 8-month follow-up compared to the control school (+9.0% mean proportion of observed children, p = <0.001) (Table 4).

**Table 4:** Objectively measured physical activity intensities and types between intervention and control schools at the three time points

**Physical activity measure**	**Baseline**			**Post-test (7-Weeks)**			**Follow-up (8-Months)**		
**Direct observation (Intensity)**	**Percentage comparison of mean children in each physical activity intensity within direct observation scans**^**#**^								
	**Intervention**	**Control**	***p***	**Intervention**	**Control**	***p***	**Intervention**	Control^*^	***p***
Sedentary behaviour (%)	7.4 (61.5)	9.7 (61.5)	0.99	6.7 (43.6)	8.0 (49.2)	*<0.01*	5.5 (40.0)	7.1 (55.2)	*<0.001*
Moderate physical activity (%)	3.5 (28.8)	4.3 (27.5)	0.61	4.3 (28.1)	4.7 (28.7)	0.80	5.3 (39.1)	3.9 (30.1)	*<0.001*
Vigorous physical activity (%)	1.2 (9.7)	1.7 (11.0)	0.66	4.3 (28.3)	3.6 (22.1)	*<0.01*	2.9 (20.9)	1.9 (14.7)	*0.01*
**Direct observation (Activity)**	**Percentage comparison of the predominant activity type within direct observation scans**^**#**^								
	**Intervention**	**Control**	***p***	**Intervention**	**Control**	***p***	**Intervention**	Control^*^	***p***
Australian rules football (%)	-	12 (5.0)	-	11 (8.8)	54 (22.5)	0.30	-	6 (1.9)	-
Baseball/Softball (%)	-	3 (1.3)	-	-	-	-	-	-	-
Basketball (%)	-	11 (4.6)	-	-	15 (6.3)	-	-	3 (1.0)	-
Cricket (%)	5 (4.0)	3 (1.3)	0.83	1 (0.8)	-	-	2 (1.6)	-	-
Down-ball (%)	-	36 (15.0)	-	-	1 (0.4)	-	-	-	-
Imaginative play (Fixed equipment) (%)	-	69 (28.7)	-	-	70 (29.2)	-	-	76 (24.4)	-
Imaginative play (No equipment) (%)	7 (5.6)	12 (5.0)	0.95	7 (5.6)	7 (2.8)	0.79	4 (3.2)	6 (2.6)	0.96
Imaginative play movable/recycled materials (%)	-	-	-	66 (52.8)	-	-	30 (24.0)	-	-
Construction with recycled materials (%)	-	-	-	16 (12.8)	-	-	33 (26.4)	-	-
No identifiable activity (%)	59 (47.2)	70 (29.2)	0.04	9 (7.2)	37 (15.4)	0.52	7 (5.6)	78 (25.0)	0.25
Play with loose sports equipment (%)	-	-	-	-	9 (3.8)	-	-	40 (12.8)	-
Racquet sports (%)	10 (8.0)	4 (1.7)	0.66	-	-	-	-	-	-
Sandpit play (%)	12 (9.6)	-	-	-	33 (13.8)	-	6 (4.8)	39 (12.5)	0.58
Soccer (%)	32 (25.6)	18 (7.4)	0.18	15 (12.0)	14 (5.8)	0.56	43 (34.4)	62 (19.8)	0.09
**Total lunchtime target setting scans (%)**	125 (100)	240 (100)	-	125 (100)	240 (100)	-	125 (100)	320 (100)	**-**

^#^The *p* values are based on Chi-square test for comparing proportions between intervention and control schools; ^*^Two more lunchtime target defined areas were introduced at the control school during follow-up; Intervention school lunchtime = 30 minutes; Control school lunchtime = 45 minutes; Direct observation utilised SOPLAY [61].

The most predominant PA type observed at the intervention school during baseline were recorded as ‘no identifiable activity’, ‘soccer’ and ‘sandpit play’ (Table 4). However, after the LEAP intervention was introduced, students within the intervention school were using the movable/recycled materials as the predominant activity at the 7-week post-test and 8-month follow-up for ‘imaginative play with movable/recycled materials’ and ‘construction with movable/recycled materials’. The other predominant PA during post-test and follow-up were ‘soccer’ (post-test and follow-up) and ‘Australian Rules Football’ (post-test). In contrast, the predominant PA types children engaged in at the control school were ‘imaginative play with fixed equipment’ (post-test and follow-up), ‘soccer’ (follow-up), ‘sandpit play’ (post-test and follow-up) and ‘Australian Rules Football’ (post-test).

### Enjoyment of physical activity and lunchtime play activities

A significant treatment effect from the LEAP intervention in the intervention school compared to the control school was identified from baseline to the 7-week post-test for children’s mean enjoyment of PA (+0.32 adjusted mean change, 95% CI = 0.04-0.61, p = 0.03), and enjoyment of intra-personal play activities (+0.24 adjusted mean change, 95% CI = 0.004-0.48, p = 0.045). There were no significant treatment effects from the intervention on children’s enjoyment of physical environment/policy level factors associated with lunchtime play activities throughout the school year. Similarly, there was no significant overall interaction effect on children’s enjoyment of PA and lunchtime play activities (Table 3 and Figure 2).

### Quality of life

A significant treatment effect from the LEAP intervention in the intervention school compared to the control school was identified from baseline to the 7-week post-test for children’s mean physical health scale of QOL (+4.61 adjusted mean change, 95% CI -2.42-11.64, p = 0.05). There were no significant treatment effects identified in the intervention school children’s mean psychosocial scale QOL and mean overall QOL compared to the control school (Table 3 and Figure 2), however trends suggest a treatment effect of borderline significance from baseline to post-test (7 weeks) (Table 3). There was no significant overall interaction effect on children’s mean QOL scores.

There were no significant (p > 0.05) age or gender effects from the LEAP intervention throughout the school year for any of the objective and self-report measures; data not reported.

## Discussion

The study extends the work conducted previously in this area with younger children [17,45,49,50] to examine the impact of this simple, cost-effective school playground intervention (or use of movable/recycled materials) targeting a whole school (5-12-years-old) with the measurement of additional PA and health outcomes. The primary outcome variable of the LEAP intervention was PA, individually and objectively measured by pedometers in children aged 5-12-years-old. The results reveal that the LEAP intervention had a significant overall interaction effect on children’s pedometer-determined PA (e.g. steps per minute, distance per minute). Pedometer-determined PA remained significantly elevated in the short-term, but to a lesser extent at 8-months. Despite the statistical reduction in steps and distance, higher intensity physical activity was evident, therefore this could be related to greater proportions of children constructing play areas and playing with more purpose within and around the constructed spaces vigorously at the 8-month follow-up, rather than accumulating steps moving around the grass field. Contrasting previous studies [68,69], the intervention school children’s PA levels increased when temperatures were cooler during the post-testing and decreased during follow-up when temperatures became warmer. Despite a steep incline on the grass field (increased difficulty to accumulate steps), students within the intervention school were consistently above children’s school lunchtime steps per minute from a United States (US) PA study (53 mean steps/min) [24], yet the control school were below this mark. The lower PA of the control school could be related to the presence of several fixed playground equipment, which encourage climbing, swinging and sliding, rather than locomotor movements. Although loose sports equipment was made available to the control school children, fixed playground equipment provides no opportunity to move objects around and is an area, along with weather influences, warranting further examination.

Consistent with findings from a similar intervention targeting 5-7-year-old children (n = 223) [50] and previous studies [24,25,27], which have reported children are engaging in high proportions of MVPA, SOPLAY measurements revealed that over 50% of children at the intervention school observed at both the 7-week post-test and 8-month follow-up were engaged in MVPA during school lunch breaks. A significantly higher proportion of children in the LEAP intervention were also observed undertaking vigorous intensity PA compared to children in the control school 7-weeks and 8-months after baseline. Although these measurements were at the playground level and represented the proportion of children engage in different PA intensities; there may be potential for children to reach the recommended guidelines for MVPA per day using these movable/recycled materials. The steps and distance measurements declined from baseline to follow-up in the intervention school, yet the significant overall interaction effect on the intervention school children’s accumulated steps and distance could reflect more options being present within the school playground [41] via the movable/recycled materials. These findings also highlight that movable/recycled materials can be used as a potentially sustainable strategy to promote children’s PA over an 8-month period and has the potential to engage all levels of primary school children.

The diversity and evolving play in the school playground environment is evident within this study with the dominant PA type imaginative play with the movable/recycled materials during post-test and building and construction during follow-up. It has been suggested that children enjoy choice in their playground activities [13] and our data supports this suggestion. Movable/recycled materials are suggested to stimulate creativity and diversity to children’s play and provide active play experiences by facilitating pushing, pulling and lifting and the construction of structures (e.g. cubby houses, rockets, ships) whilst engaging in social interaction and problem-solving [45]. Interestingly, despite soccer being a dominant PA type in school playgrounds [70], children were seen incorporating the movable/recycled items into their sport e.g. as football goals. The safety policies identified via field notes including stacking restrictions, not striking others and the removal of damaged equipment may also have helped facilitate an environment in which children could freely and safely engage in PA. The findings of the present intervention study on children’s PA provide further evidence of the benefits of implementing movable/recycled materials during school breaks [45].

The secondary outcome variables included enjoyment of PA, enjoyment of lunchtime play activities and QOL in those children aged 8-12-years-old. Similar to previously reported PA research [22,31], we found short-term treatment effects for children’s enjoyment of PA and intra-personal play activities and QOL (physical health scale). Counter to our predictions we did not find any longer term impacts of the intervention on enjoyment and QOL (psychosocial health scale) measures. A possible explanation for the lack of significant overall interaction effects being identified for enjoyment of inter-personal, enjoyment of physical environment/policy level lunchtime play activities, psychosocial scale QOL and overall QOL could simply be that baseline measurements were undertaken at the beginning of the school year, as children may have been content with or enjoying returning to school play activities during the warm weather after a long summer break. As enjoyment and QOL data were rated high at baseline, a ceiling effect may have been evident, with little margin for mean enjoyment or QOL increases after introducing the LEAP intervention. Furthermore, children’s mean enjoyment and QOL scores at the intervention school were higher than previous studies using the LEAP questionnaire [56] and Peds QL 4.0 [71,72] with similar aged primary school children. Further administration of the enjoyment questionnaires throughout the school year may help elucidate the impact of the intervention on enjoyment further [15].

Strengths of the study include responding to a range of recommendations for school-based PA interventions [51]; including the use of valid objective PA measures, examining a mediator of PA (e.g. enjoyment), measuring multiple dimensions of school children’s PA participation and a long-term follow-up. As all PA measures have limitations, it is important that a combination of measures were used to assess children’s school-based PA [55]. The present study fills a gap in the literature by examining children’s enjoyment of PA within a school lunchtime context [56]. Furthermore, no PA intervention study we are aware of targeting primary school breaks has examined children’s PA distances covered or QOL outcomes. Evaluating an intervention’s potential to positively influence social-ecological levels of influence on children’s PA is important to enhance long-term PA outcomes [52,70]. The long-term patterns of PA identified from the LEAP intervention can help inform public policy and debate regarding school playground environments during school breaks [73]. Understanding children’s health behaviours within the school context is important [27,28], however little research has examined how children’s PA and play behaviour can change over time in response to a modified school playground. In addition, little research has used the PACES questionnaire in younger age groups since being validated in primary school children [64]. This is also the first school lunchtime intervention we are aware of to use the context-specific LEAP questionnaire to evaluate an intervention targeting school lunch breaks [56].

As many playgrounds are designed and installed without consultation with children [13], providing children with the materials to facilitate and direct their own play reflects growing educational trends to provide student, rather than teacher-directed PA opportunities [14]. Unstructured, active play allows children to understand their world and develop skills, therefore school playground environments should be developed in a manner that enhances development and physical functioning of children [7]. With the modern demands on schools to equip children with skills to be physically active, the LEAP intervention could be implemented without placing increased burden on already busy teaching staff. The LEAP intervention provides a cost-effective and potentially sustainable key public health strategy that could be used to develop children’s PA within the ‘informal’ curriculum of school breaks.

### Limitations

There were several limitations to the study. Firstly, it should be acknowledged that the effects of the intervention were intended to be assessed 13-weeks after baseline as well as at 7-weeks, however due to the highest rainfall for the region on record, investigators could only complete data collection at two time points after baseline (7-weeks and 8-months). As the data was collected during school lunch breaks, the findings may not be reflective of PA during morning or afternoon school breaks. The intervention school did not contain any regular fixed playground equipment and it is possible that children may have embraced the movable/recycled materials more readily than a school with a conventional school playground. Although conventional, fixed playground equipment has been reported to restrict diverse play opportunities [42], future research could examine the PA of school children with access to both movable/recycled materials and conventional, fixed playground equipment. As the LEAP intervention was implemented within a single primary school to take advantage of a real world opportunity (newly developed primary school campus with no pre-existing fixed playground), there was no adjustment for cluster and the findings from the study should not be generalised. Due to both schools implementing a ‘no hat, no play’ policy as part of being sun-smart schools, sex-specific identification was unable to be determined via direct observation. Furthermore, we were unable to objectively measure the physical benefits of lifting, dragging and carrying movable/recycled materials around the playground, despite multiple dimensions of PA being accounted for [55]. As school lunch breaks at the intervention school totalled 30 minutes, compliance with the national PA guidelines of 60 minutes of MVPA was unable to be assessed. Moreover, the mean maximum temperature at the control school during follow-up was significantly lower than the intervention school and the lower mean age also resulted in a smaller sub-sample of the intervention school completing self-report measurements. The control school also installed two small play areas (synthetic soccer court and an empty natural play area) during follow-up, however this can be expected within a long-term research intervention targeting a real world setting such as a school. The introduced spaces at the control school had little impact on children’s PA behaviour.

## Conclusions

This LEAP intervention was designed as a feasible, simple and innovative approach to increase PA within the school playground and had a significant overall intervention effect on children’s objectively measured PA, including mean steps and distance per minute in the intervention school compared to the control school for the three time points. A short-term treatment effect was revealed in the intervention school compared to the control school for children’s physical health scale QOL, enjoyment of PA and enjoyment of intra-personal play activities after 7-weeks. However, there were no significant effects from the intervention on children’s enjoyment of inter-personal level play activities, enjoyment of physical environment/policy level play activities and overall QOL. The intervention school children spent significantly higher proportions within specified playground target areas in more vigorous PA intensities than the control school children at both 7-weeks and 8-months after baseline. Direct observation of the intervention school children’s lunchtime break activities throughout the school year revealed that the intervention facilitated evolving play opportunities, including imaginative play with the movable/recycled materials (predominant PA type during post-test) which eventually evolved into a building and construction phase with the materials (predominant PA type during follow-up). The positive PA, enjoyment and QOL outcomes from this simple, low-cost intervention could be used to inform the development of future intervention programs using movable/recycled materials on a wider scale within primary school settings.

## Abbreviations

LEAP: Lunchtime Enjoyment of Activity and Play; LPA: Light Physical Activity; MPA: Moderate Physical Activity; MVPA: Moderate-Vigorous Physical Activity; PA: Physical Activity; PACES: Physical Activity Children’s Enjoyment Scale; PE: Physical Education; QOL: Quality of Life; SOPLAY: System of Observing Play and Leisure Activities in Youth; VPA: Vigorous Physical Activity.

## Competing interests

The authors declare that they have no competing interests.

## Authors’ contributions

BPH contributed to the conception, design and implementation of intervention methodology, acquisition of data, analysis and interpretation of data, drafting, critical review and final submission of the manuscript. ACB contributed to the design of intervention methodology, analysis and interpretation of data, drafting and critical review of the manuscript. SU contributed to the analysis and interpretation, drafting and critical review of data. AT contributed to the conception, design of intervention methodology, acquisition of data, analysis, drafting and critical review of the manuscript. All authors read and approved the manuscript.

## Pre-publication history

The pre-publication history for this paper can be accessed here:

http://www.biomedcentral.com/1471-2458/14/164/prepub
